# Evaluation of Isfahan’s Dental Students’ Awareness about Preventive Dentistry

**Published:** 2014-03

**Authors:** F. Nilchian, Sh. Kazemi, M. Abbasi, F. Ghoreishian, M. Kowkabi

**Affiliations:** a Dept. of Dental Public Health, Member of Torabinejad’s Research Center, School of Dentistry, Isfahan University of Medical Sciences, Isfahan, Iran.; b Dental Student, Student Research Committee, School of Dentistry, Isfahan University of Medical Sciences, Isfahan, Iran.

**Keywords:** Preventive Dentistry, Knowledge, Education, Dentist

## Abstract

**Statement of Problem:** The modern dentistry approach is moving toward preventive dentistry, an approach that has decreased the prevalence of caries within the past decades. Since some reports imply that dentists are not knowledgeable enough in this issue.

**Purpose:** This study aimed to evaluate the attitude and awareness of future dental graduates toward preventive dentistry regarding gender and educational characteristics.

**Materials and Method: **This cross-sectional study was performed on one hundred and forty questionnaires which were distributed among dental students of Isfahan province, Azad university of Khorasgan, Iran. Data regarding the level of awareness of dental students about preventive dentistry were recorded and analyzed by using the mean and standard deviations on scores as appropriated.

**Results:** Majority of dental students were aware about the role of sugar in caries process, while only a minority were aware about the role of fluoridated toothpaste and brushing method in caries prevention. Most of the students (82.1%) were among the group with medium level of awareness. Compared with their female counterparts, male students had more knowledge about fluoride efficacy and general hygiene role in caries process.

**Conclusion:** Dental students of  the Isfahan State University and Azad University of Khorasgan had acceptable levels of awareness about the role of sugar and fluoridated water in caries process, but were not aware enough about the role of fluoridated toothpaste in preventing dental caries.

## Introduction

Oral and dental hygiene may be improved in society through enhancing dentists’ knowledge and attitude regarding this issue [1]. Dentists also influence oral hygiene associated behaviors of the patients. As dental science develops, dentists are forced to update their knowledge using the latest scientific sources available. The treatment plan of each dentist is influenced by his attitude toward treatment methods and their clinical significance [2-3]. The issue of prevention in dentistry has reduced the prevalence of caries in recent decades and it will constitute the most important part of dental services in near future [4-5].

Preventive dentistry is among the priorities of the World Health Organization (WHO) for oral health promotion [6], the reason that dentists’ knowledge and attitude should be conducted in the same path. No clear and reliable information is in hand about the level of dentists’ knowledge on preventive dentistry [7]. University students in many countries have a significant role in public life and can be leaders in the future. A Comparative study of oral health attitudes and behavior among dental students in Britain and China shows that as future health professionals, they should have a comprehensive program including self-care regimens. Preventive dental science curricula need to be changed to incorporate oral self-care regimens not only in clinical practices, but also in academic and public policy [18]. Although dentists seem to be aware of this issue, reports indicate insufficient awareness on preventive dentistry [1, 8-11]. Considering the significant role of preventive dentistry in today’s dentistry, studies might be designed to evaluate the knowledge level of current dental students on preventive dentistry so that suitable policies can be made to enhance dentists’ awareness. Searching different electronic databases, no studies were found to have evaluated Iranian dental students’ preventive knowledge. Therefore, the present study aimed to evaluate the knowledge and attitude of future dentistry graduates about preventive dentistry according to their age, gender and educational characteristics.

**Table 1:** The average mean of students’ score in response to the 9 statements separated by gender

		**Total**	**Male**	**Female**
Caries related	Frequency of sugar consumption	4.77	4.73	4.79
	Sugar-free chewing gum	4.37	4.34	4.39
	Xylitol	3.69	3.83	3.61
	Regular check-ups	3.27	3.24	3.29
	The importance of brushing technique	2.70	2.77	2.67
Fluoride related	Fluoride is the most important factor for tooth susceptibility to decay.	4.30	4.38	4.25
	Fluoridation of drinking water	3.60	3.57	3.62
	Fluoride toothpaste	2.15	2.14	2.16
Oral and dental hygiene	Regular brushing	4.25	4.34	4.19
Total** **		33.14	33.38	33.01

## Materials and Method

This descriptive cross-sectional study was done on dentistry students in Isfahan, Iran. Out of 140 dental students participating in this study, 94 were chosen from Isfahan University of Medical Sciences and 46 from Azad University of Khorasgan. Each student was given a questionnaire including age, gender, year of entrance to the university, type of university and whether the student has passed the dental public health course or not. In order to evaluate the level of knowledge of students on preventive dentistry, the questionnaire included 9 related statements (Appendix 1), adapted from a current article and a PhD thesis from Helsinki University of Helsinki in Finland [12-13]. The answers were scored on a 5-point Lickert scale ranging from 1 (completely disagree) to 5 (completely agree). The total score of each questionnaire shows how knowledgeable the participant is about preventive dentistry. The participants who did not answer three or more statements were excluded. In case of no answer for less than three statements, the "I don't know" answer was considered. Data were analyzed using SPSS software, version 12. Descriptive statistics (mean and standard deviation) were used as appropriated.  Data analysis was done according to age, gender and whether or not the dental public health course was passed. The P value<0.05 was adopted as significant level.

## Results

Dental students’ knowledge about preventive dentistry was measured using a 9-statement questionnaire and scored based on Lickert scale (Appendix 1). The total score of each questionnaire indicated the participant’s knowledge. Table 1 shows the mean degree of male and female students’ knowledge regarding the gender. Based on the distribution of total scores, participants were divided into 3 groups: poor knowledge (total score<27), intermediate knowledge (total score 28-36) and high knowledge (total score 37-45).

Table 2 shows the level of students’ knowledge regarding the year of entrance in university, gender, type of university and passing the dental public health course.

**Table 2:** The students’ knowledge level separated by year of entrance in university, gender, type of university, and passing the dental public health course

		**High** **(%)**	**Moderate** **(%)**	**Low ** **(%)**	**Total** **(%)**
Entrance year	4th year	10.3	88	1.7	58
	5th year	23	74.3	2.7	39
	6th year	16.3	81.4	0.3	43
Gender	Female	14.28	82.4	3.32	91
	Male	18.4	81.6	0	49
Dental public health course	Passed	16.8	81.6	1.6	125
	Did not pass	6.6	86.8	6.6	15
Total		15.7	82	2.1	140

The level of student s’ knowledge about the three fundamental issues of preventive dentistry, in proportion to the level of knowledge, was measured and recorded in table 3.

**Table 3:** Students’ knowledge level about the 3 fundamental issues of preventive dentistry in proportion to the level of the knowledge

	**High Knowledge**	**Moderate Knowledge**	**Few Knowledge**
Caries related	27.9	65	7.1
Fluoride related	37.9	62.9	0
Dental and oral hygiene	32.9	69.9	4.3

The students were well informed about the effect of sugar in caries process, but poorly informed about the use of toothpaste containing fluoride compared with brushing technique for caries prevention (Question 5). The knowledge of male students in the statements related to the caries process, fluoride efficacy and tooth, and general hygiene role was higher than their female counterparts. The majority of students (82.1%) expressed an intermediate level of knowledge. These students were better informed in all three fields of preventive dentistry than other students. The average score of Khorasgan dental student was 32.19 and the average score of students of Isfahan University of Medical Sciences was 33.6; that can be due to the small sample size chosen from Khorasgan University.

## Discussion

The results of this study show that dental students of Azad University of Khorasgan and University of Isfahan Medical Sciences had sufficient and acceptable level of knowledge about the effect of sugar, sealant, and fluoridated water on tooth caries and caries prevention; however, they were not informed about the role of fluoridated toothpaste on caries prevention and did not have enough experience in diagnosing the depth of caries penetration. Male students seemed to be more informed about preventive dentistry than their female counterparts.

Khami et al. evaluated the oral health behavior of Iranian dental students with respect to their gender, background characteristics, preventive care knowledge, and attitudes towards preventive dentistry [19]. They concluded that education and training in preventive measures should be effective enough to overcome background characteristics. Therefore, oral health behavior (OHB) can be improved in Iranian dental students [19]; that are similar to the result of this study. Based on the current study, the majority of the students who have passed dental public health course were classified as those with high knowledge compared with those who had not passed this course.

Evaluating preventive knowledge of Jordanian students showed that dental preventive courses had improved dental students’ oral health awareness and attitudes. Therefore Barrieshi-Nusair et al. concluded that preventive dentistry courses should be taught early in the dental curriculum of pre-clinical years [13].

There are contradictory theories about the efficacy of fluoride in caries prevention [14]. Based on new theories, after brushing the teeth, mouth rinsing should be limited to a bare minimum so that the fluoride in the toothpaste can most effectively prevent the caries [15].

After their graduation, dentists' knowledge about preventive dentistry moderates as time passes [1, 11].

If suitably planned, post-graduation education may enhance dentists’ knowledge [2]. This shows the need for educational programs after graduation, however, it has had no effect on Mongolian dentists’ knowledge [7. This may be due to the weak educational content and inappropriate teaching method. The difference between dentists’ awareness in preventive dentistry field may be due to different treatment planning approaches in different countries [7]. The results of the present study are in agreement with the ones performed in Korea and Mongolia; both of which reported little knowledge about the effect of fluoride on preventing caries [11, 16]. While in Finland with a long historical background in preventive dentistry, dentists are well informed about the significant role of fluoride in caries prevention [17]. Therefore, the current educational concepts should magnify the role of fluoride in caries prevention. One of the limitations of the study was that in Khorasgan University, the dental public health course is presented in the last educational year in an intensive pattern which causes another limitation for performing this study. The evaluating questions used in this study included basic information and are comparable with the studies performed on Korean’s and Mongolian’s dentists [11, 16]. More accurate and detailed questions in this field should be made in future studies.

## Conclusion

Dental students of Isfahan State University and Azad

University of Khorasgan had acceptable levels of awareness about the role of sugar and fluoridated water on caries process, but were not aware enough about the role of fluoridated toothpaste in preventing dental caries.
